# Determining known-group validity and test-retest reliability in the PEQ (personalized exercise questionnaire)

**DOI:** 10.1186/s12891-019-2761-3

**Published:** 2019-08-14

**Authors:** Isabel B. Rodrigues, Jonathan D. Adachi, Karen A. Beattie, Arthur Lau, Joy C. MacDermid

**Affiliations:** 1 University of Waterloo, 200 University Avenue West, BMH 1102, Waterloo, Ontario N2L 3G1 Canada; 2 Department of Medicine, McMaster University, 25 Charlton Ave. E Room 501, Hamilton, Ontario L8N 1Y2 Canada; 3 St. Joseph’s Healthcare Hamilton, 25 Charlton Ave. E Room 501, Ontario, Hamilton L8N 1Y2 Canada; 4 The University of Western Ontario, 930 Richmond St, London, ON N6A 3J4 Canada; 5 Hand and Upper Limb Center Clinical Research Lab, 930 Richmond St, London, ON N6A 3J4 Canada

**Keywords:** Exercise adherence, Barriers, Facilitators, Preferences, PEQ, Validity, Reliability

## Abstract

**Background:**

To determine the known-group validity, a type of construct validity, and the test-retest reliability of a newly developed tool, the Personalized Exercise Questionnaire (PEQ), that assesses the barriers, facilitators, and preferences to exercise in individuals with low bone mass and osteoporosis.

**Methods:**

A comparative design was used to assess known-group validity and a test-retest design to examine the reproducibility. Ninety-five participants with low bone mass and osteoporosis were recruited from an outpatient clinic in Hamilton, Ontario. The questionnaire was administered to 95 participants at baseline and a subset of 42 participants completed the survey again one week later. The known-group validity of the PEQ was determined using four hypotheses that compared two known groups based on employment level, age, socioeconomic status, and physical activity level. The reproducibility of individual responses was analyzed using the Kappa Coefficient (κ).

**Results:**

There was known-group validity for three of the four hypotheses. Test-retest reliability scores ranged from no agreement to almost perfect agreement; seven items had almost perfect agreement (κ: 0.81–1.00), 12 substantial agreement (κ: 0.68–0.74), six moderate agreement (κ: 0.56–0.60), two fair agreement (κ: 0.36–0.40), one slight agreement (κ = 0.23) and one no agreement (κ = − 0.03).

**Conclusion:**

Preliminary support for the usefulness of the PEQ is indicated since the majority of the items had at least substantial agreement and known-group validity was moderately supported for some items.

**Trial registration:**

This study was retrospectively registered with ClinicalTrials.gov, NCT03125590, on April 24, 2017.

## Background

Regular physical activity is an important component for maintaining a healthy lifestyle and an essential factor for prevention of osteoporosis. Yet, despite the well-known benefits of regular activity, surveys found more than 60% of adults do not engage in regular exercise and 31% do not participate in any activity [1]. A systematic review published by our group reported adherence rates to exercise in people with osteoporosis to be between 52 to 100% [2]. One method that might increase exercise adherence is to understand the factors that affect the motivators, barriers, and preferences to physical activity and employ methods to leverage facilitators and preferences and limit barriers to create customized exercise programs [1]. Questionnaires are the most frequently used method of data collection in the field of rehabilitation science and the most feasible option to survey large populations [3, 4]. These self-report questionnaires may be one method to collect data regarding factors that affect exercise adherence. Understanding the factors affecting exercise adherence may help develop targeted interventions that increase the quality and delivery of physical activity programs in the research setting and in clinical practice. [4]. A growing body of literature has examined levels of physical activity among different populations using self-reported questionnaires and there is an increased interest to integrate patient-reported outcomes into clinical practice [3].

Exercise is widely recommended to reduce the effects of osteoporosis, falls, and related fragility fractures and a number of systematic reviews found weight-bearing exercises help maintain or increase bone mineral density (BMD) in the hip and spine of women with low bone mass [5–8]. The effects of exercise are not only concentrated in reducing the consequences of osteoporosis but also play an important role in improving daily activities [9]. A recent systematic review found exercise also improves activities of daily living (e.g., dressing, bathing, etc.) in participants with osteoporosis [9].

We previously described the developmental process and content validity of the Personalized Exercise Questionnaire (PEQ); a self-reported survey that assesses the motivators, barriers, and patient preferences to exercise [10]. Although a previous tool (the Exercise Benefits/Barriers Scale or EBBS) exists, it does not cover some of the most frequently reported barriers in older adults such as lack of interest, lack of transportation, pain, disliking going out alone, etc. The EBBS also has minimal focus on the specific type of exercise that would be preferred, and so the PEQ was developed from a number of systematic reviews, expert advice, and participant feedback to address these issues [10]. In a previous paper, the PEQ demonstrated high content validity of individual items (I-CVI range: 0.50 to 1.00) and moderate to high overall content validity (S-CVI/UA = 0.63; S-CVI/Ave = 0.91) among healthcare providers [10]. This article describes the sequential steps in the testing of the PEQ using data collected from patients with low bone mass or osteoporosis. The purposes of this study were to describe the:

1. Cross-sectional construct validity by testing differences between two or more groups with expected differences to establish known-group validity [11];
2. Test-retest reliability of individual items of the PEQ by measuring the stability of an item’s response over time [11].

## Methods

### Ethics

The study was carried out in accordance with the Code of Ethics from the Hamilton Integrated Research Ethics Board (HiREB) and was associated with the research project administered through St. Joseph’s Healthcare Hamilton and McMaster University. The Research Ethics Board in Hamilton approved this project on February 24, 2017 (project number: 2682). This trial was retrospectively registered on April 24th, 2017 in ClinicalTrials.gov under identifier NCT03125590 and last updated on August 29th, 2017.

### Sponsor and funder role

This study was funded by the Canadian Institutes of Health Research (funding reference number: 122070 and 137,148) and the Dr. James Roth Research Chair in Musculoskeletal Measurement and Knowledge Translation award. The funders had no role in the study design, data collection and analysis, decision to publish, or preparation of the manuscript.

### The personalized exercise questionnaire

The final version of the PEQ consists of 6 domains and 38 questions (35 categorical questions and 3 open ended). Open-ended questions prompt the individual to identify up to three items that facilitate or prevent physically active and may provide a unique way of integrating more information that may not have been captured through closed-ended questions. The other 35 questions were categorized in 6 domains: 1) my support network (e.g., healthcare provider, family, or friend’s attitude toward exercise), 2) my access to exercise (e.g., location, transportation, or cost barriers), 3) my exercise goals, 4) my exercise preferences, 5) my feedback and tracking (e.g., technology use), and 6) my exercise barriers [10]. Rather than obtain a total score, a summary score for each domain should be calculated and interpreted separately since each domain score provides valuable information. For example, section one (my support network) has three questions to determine the strength of a person’s social network and can have a maximum score of 3, where “no”, “not sure”, and “not applicable” receive a score of 0 for each item, and “yes” a score of 1. If all three items are marked “yes” the score is 3, if only two are marked “yes”, the score is 2, and if only one is marked “yes” the score is 1. A score of 3 indicates a strong support network and evidence suggests that insufficient social support for exercise in older adults is a key barrier to participation in an exercise program [12]. More information on scoring can be found in Appendix A.

### Data collection

#### Study procedures

A convenience sample was sought at the St. Joseph’s Healthcare Hamilton centre. Medical records were accessed for the purpose of identifying and recruiting participants and all procedures followed the HIPAA regulations and were approved by the Hamilton Ethics board. Eligible participants were directly identified in the clinic by their rheumatologist (JA or AL) based on the following inclusion criteria: 1) able to provide informed consent, 2) ≥ 18 years old, 3) diagnosed with low bone mass or primary osteoporosis (T-score < − 1.0) at the lumbar spine or femoral neck, and 4) could comprehend, read, and write English. Participants with a cognitive impairment were excluded. Patients were recruited non-consecutively from March 13, 2017 to May 3, 2017 at the St. Joseph’s Healthcare Hamilton Charlton Campus rheumatology clinic.

Eligible participants were quickly briefed about the study by their rheumatologist (JA or AL) and potential participants who indicated they would like to hear more were introduced to the research assistant (IR) who went over the study protocol and invited them to complete the questionnaire. Willing participants then signed a consent form and completed a demographic survey and the PEQ either in the clinic or at home. Participants were asked to answer items based on their current living conditions. The majority of participants who finished the survey in the clinic were asked by IR whether they would complete the same questionnaire 7 days later. Those who agreed were given the PEQ in a self-addressed, return envelope. Participants’ records were de-identified and distinguished using Personal Identification Digits (PID). A PID was written on each form on the top left corner of the demographic survey, the PEQ, and return envelopes. Nonrespondents were contacted by telephone 30 days after their initial visit.

#### Sample size

Two sample sizes were calculated, one for the known group validity study and the other for the reliability study. A two-tailed test with a power of 80%, α = 0.05 and a dropout of 20% requires at least 114 participants for the comparison study. The sample size required to estimate the intra-rater reliability coefficient at a 0.05 level of significance and 80% power is 46 (p_0_ = 0.8; p_1_ = 0.9) [13]. A higher p_0_ indicates greater reliability, with p_0_ = 0.8 indicating the highest acceptable level of reliability [13].

### Measurement properties

A cross-sectional, comparative design was used to test the known-group validity and a test-retest design to test the reproducibility of the PEQ in participants with low bone mass or osteoporosis. All statistical analysis were computed in SPSS version 22.

#### Known group validity

This type of construct validity measures an instrument’s ability to distinguish among distinct groups [14]. Group differences were determined using the chi-square test of independence followed by post-hoc analysis. Four hypotheses were identified a priori to determine known group validity:

1. Participants working full-time are more likely to report time as a barrier to exercise [15, 16];
2. There is no difference between group-related intervention strategies amongst older adults (65 and older) and middle aged adults [17];
3. Participants from a lower socioeconomic status (SES), less than <$20,000, are more likely to report finances as barrier to exercise [16, 18, 19];
4. Participants with a safe place to exercise (i.e. proper space to exercise, dry and clean floors, good lighting, etc.) are more likely to be physically active [15];

Chi-square tests were used since variables were nominal and the phi coefficient (also known as Choen’s *w*) was used to calculate effect size. A phi coefficient between 0.10 to 0.30 is considered small, 0.30 to 0.50 moderate, and greater than 0.50 large [20]. Question 34 was used to evaluate hypotheses 1 and 4, question 22 hypothesis 2, and question 7 hypothesis 3. In question 7, items marked “yes” were considered safe while “no” and “not sure” considered unsafe.

#### Test-retest reliability

This is a measure of stability of an instrument over time through repeated testing and is assessed at two different time points. Participants were given the PEQ at baseline (day 1) and then asked to repeat the same survey again 1 week later (day 7). Seven days were chosen to give participants enough time so they would not remember their answers from the initial assessment. Although the Intraclass Correlation Coefficient is effective for quantifying the reproducibility of continuous data, the items in the PEQ are nominal or ordinal and was not designed to have a summative score. So kappa coefficient of Cohen, also known as Cohen’s kappa, and weighted kappa were used to estimate the chance-corrected agreement as a measure of test-retest reliability. Cohen’s kappa was used for domains one, two, four, five and six, while weighted kappa for section three, which used ordinal answers. Since kappa can be problematic to interpret when responses have little variation, percentage agreement was also calculated. Kappa can range from − 1 to + 1, where 0 represents the agreement occurring by random chance and 1 represents perfect agreement between answers [21]. A kappa < 0 indicates no agreement, 0.01–0.20 none to slight, 0.21–0.40 fair, 0.41–0.60 moderate, 0.61–0.80 substantial, and 0.81 to 1.00 almost perfect agreement [21]. Percentage agreement was considered high if it exceeded 75%, moderate between 40 and 75% and low if less than 40%.

#### Response handling

Responses were entered into excel where columns represented distinct questions and each row a participant. For example, section one (my support network) had three columns corresponding to questions 1, 2, and 3 and each answer was assigned a numerical value such that “no” corresponded to “1”, “not sure” to 2, “yes” to 3, and “not applicable” to 4. If answers were missing, the excel cell would be left blank and removed from analysis. Questions with multiple answers such as those in sections 4, 5, and 6 were recorded differently. For example, section 4, question 19 (“where would you like your exercise program to be”) has 6 choices, and if “gym” and “community centre” were checked, these marked answers would be assigned the value “1” while if unmarked, a value “0”. So the excel cell for column 19 would be recorded as a binary code “010100”. In this specific case, since there are 6 choices, there are 2^6^ = 64 possible binary codes and each code is assigned a decimal value such that “000000” would correspond to “1”, “000001” to “2”, “000010” to 3, …., and “111,111” to 64. Conversion of binary codes to single numerical values make it easier to compare results for test-retest.

## Results

### Descriptive characteristics

The PEQ was administered to 114 participants and 95 questionnaires and 42 test-retest questionnaires were completed. Seven individuals declined to participate. General demographic characteristics are summarized in Table 1. The mean age of the participants was 66.1 (9.88) with the majority between 50 to 79, specifically, 4% less than 50, 38% between 50 to 64, 43% between 65 to 79, and 15% 80 and over. Fifty-six participants were retired, 22 worked full-time, 9 part-time, and 8 did not work due to disability. Sixty-eight participants self-identified as physically active, 20 as “not active”, and 7 were not sure if they were physically active. At the time of administering the PEQ, 87 participants were on medications, most in combination with vitamin D and calcium. The majority of participants were on a denosumab such as Prolia (64%) or a bisphosphonate such as Actonel (23%). Thirty-nine participants were diagnosed with osteoporosis of the spine and 56 with low bone mass of the spine; 25 with osteoporosis of the hip and 70 with low bone mass of the hip. All patients were reported to be non-smokers. Eleven participants used mobility devices, 4 used a cane, 3 a cane and a walker, 2 a walker, 1 a wheelchair and a walker, and 1 a wheelchair. There were no differences in terms of age, gender, SES, and T-scores of the hip or spine (*p* > 0.05) between groups that completed the PEQ in clinic and those that competed it at home. More than half of the participants had a prior fracture, some had multiple fractures.

**Table 1:** Demographic characteristics of participants

Characteristics	Number of participants
Age (years)	< 50 = 4
	50–64 = 36
	65–79 = 41
	> 80 = 14
Females (n (%))	87 (92%)
Marital Status	Single = 9
	Married = 61
	Divorced = 11
	Common-law = 4
	Domestic partnership = 1
	Widow/widower = 9
Highest education achieved	Grade school = 6
	High school = 25
	College = 30
	University = 34
Neighbourhood classification	City = 46
	Suburban = 32
	Rural = 15
	Town = 2
Household income level	< $20,000 = 20
	$20,000 – $49,000 = 40
	$50,000 – $79,000 = 17
	$80,000 – $99,000 = 8
	> $100,000 = 5
	No response = 5
Employment level	Full-time = 22
	Part-time = 9
	Retired = 56
	Not working due to disability = 7
	Not working = 1
T-score, hip (SD)/spine (SD)	−1.89 (0.74) / - 1.87 (1.28)
Prior fracture	61 (64%)

### Known-group validity

The results of the chi square test of independence to determine known group validity are presented in Table 2. Values with *p* < 0.05 were considered statistically significant. The first, second, and fourth hypotheses demonstrate high validity for questions 34 and 22, however there was no support for question 7 (hypothesis 3).

**Table 2:** Chi Square values and effect size for known-group validity

Hypothesis	Chi Square Raw Values			Chi Square Value (x^2^)	Interpretation of chi square value	Effect Size (phi)
Participants working full-time are more likely to report time as a barrier to exercise		Full-time	Not Full-time	31.34*	Accept hypothesis	1.15*
	Time-barrier	19	15			
	Time- not a barrier	3	58			
There is no difference between group-related intervention strategies between older adults and middle aged adults		< 65 years	65 + years	0.00	Accept hypothesis	0.99*
	Exercise Alone - Yes	19	26			
	Exercise Alone - No	21	29			
Participants from a lower SES are more likely to report finances as a barrier to exercise		< 20 (SES)	20 + (SES)	0.01	Reject hypothesis	0.92*
	Finances- barrier	3	10			
	Finances – not a barrier	17	60			
Participants with a safe place to exercise are more likely to be physically active		PA –Yes	PA - No	6.25*	Accept hypothesis	1.04*
	Safe Place- Yes	64	0			
	Safe Place - No	24	3			

* indicates significant *p* < 0.05

### Test-retest reliability

Absolute agreement and Cohen’s kappa were calculated for each item in sections 1, 2, 4, 5, and 6 and a weighted kappa for each item in section 3. The majority of items had substantial agreement (19 items) with 10 items had moderate agreement or less. Results are summarized in Tables 3 and 4. Reliability was calculated using 42 surveys.

**Table 3:** Cohen’s Kappa calculations for sections 1, 2, 4, 5 and 6 (*n* = 42)

Item	Absolute Agreement (%)	Cohen’s Kappa	Interpretation	
Section 1:			Absolute Agreement	Cohen’s Kappa
1	0.98	0.95*	High	Almost perfect agreement
2	0.78	0.56	High	Moderate agreement
3	0.83	0.53*	High	Moderate agreement
Section 2:				
4	0.95	- 0.03	High	No Agreement
5	0.76	0.23	High	Slight agreement
6	0.93	0.84*	High	Almost perfect agreement
7	0.98	0.90*	High	Almost perfect agreement
8	0.83	0.64*	High	Substantial agreement
9	0.90	0.69*	High	Substantial agreement
Section 4:				
19	0.60	0.56*	Moderate	Moderate agreement
20	0.83	0.76*	High	Substantial agreement
21	0.81	0.73*	High	Substantial agreement
22	0.69	0.59*	Moderate	Moderate agreement
23	0.57	0.53*	Moderate	Moderate agreement
24	0.69	0.60*	Moderate	Moderate agreement
Section 5:				
25	0.88	0.74*	High	Substantial agreement
28	0.93	0.85*	High	Almost perfect agreement
29	0.88	0.75*	High	Substantial agreement
Section 6:				
32	0.71	0.69*	Moderate	Substantial agreement
33	1.00	1.00*	High	Almost perfect agreement
34	0.71	0.69*	Moderate	Substantial agreement
35	0.79	0.66*	High	Substantial agreement

* indicates significant p < 0.05

**Table 4:** Linear weighted Kappa calculations for section 3 (n = 42) * CI 95%

Item	Weighted Kappa	Interpretation
Section 3:		
10	0.68*	Substantial agreement
11	0.40*	Fair agreement
12	0.36*	Fair agreement
13	0.68*	Substantial agreement
14	0.81*	Almost perfect agreement
15	0.79*	Substantial agreement
16	0.86*	Almost perfect agreement

* indicates significant p < 0.05

A little less than a third of participants (31%) were inconsistent with their answers for question 11, and 17% for question 12. From the participants that answered question 11 differently from round 1 to 2, 38% of this 1/3 selected “very important” the first time and “somewhat important” the second, and 15% of this same 1/3 selected “very important” the first time and “not important” the second. More than half of participants that changed their answers decided that this goal was no longer important compared to other goals. Similarly, more than half of participants (57%) of those that answered question 12 differently (17%) indicated an option of higher importance the second time.

### Flooring and ceiling effects

Flooring and ceiling effects were determined by calculating the number of people who appear in the lower and upper 10% of the total score (see Table 5). Only domains 1, 2, 3 and 5 are summative and were included in this analysis. The last column, “N”, indicates the number of participants. If at least one item was not answered in a domain, that individual’s entire response for that domain was removed from the analysis.

**Table 5:** Floor and ceiling effects in the PEQ

Section:	Flooring (%)	Ceiling (%)	N
1	14.0	28.0	93
2	1.1	28.3	92
3	0	44.9	89
5	32.6	34.8	89

## Discussion

There is now strong evidence that regular exercise can improve health related outcomes in adults and older adults and there is emerging data for significant psychological and cognitive benefits accrued from regular exercise [22]. The Canadian Physical Activity Guidelines recommend adults aged 18 to 64 accumulate at least “150 minutes of moderate–to-vigorous intensity aerobic physical activity per week and at least 2 days per week of muscle and bone strengthening activities” [23]. However, in 2013 just over two in ten Canadian adults ≥18 years of age met the physical activity guidelines [24]. To gain a better understanding of the issues associated with physical inactivity, this study aimed to validate and determine the reliability of the PEQ as a tool to assess the barriers and the facilitators to exercise.

Using the PEQ to understand the factors that influence exercise behaviours may be one method to increase adherence and create a more individualized exercise program. Despite the challenges in validating a questionnaire that captures different facilitators, barriers, and preferences we were able to provide preliminary support that the PEQ is able to provide valid and reliable information on these aspects. Validity has to be established through multiple evaluations of content, construct, and where possible criterion validity. In a previous paper, we described the development of the PEQ and the need to create this tool to address the gap in the literature [10]. Known-group validity is a form of construct validity where hypotheses are pre-specified and then tested to reflect whether a tool is able to differentiate where differences are expected a priori. Where a statistical difference is found, it supports the validity of the tool and where differences are not significant, either the tool/item is flawed, the hypothesis flawed, or the power inadequate.

The first hypothesis tested whether participants working full-time are more likely to report lack of time as a barrier to exercise. This premise was strongly supported in the results and the phi coefficient (effect size) suggested a strong difference between these two groups supporting the validity of question 34. Past studies report a lack of time is a major barrier to physical activity participation [2, 25] but one study found lack of time appears to be an excuse rather than a true reason for not being active [20]. Approximately 28 h of leisure time were spent per week doing sedentary activities such as watching television, reading for pleasure, napping, and sitting quietly [20]. This item may help clinicians identify working individuals who have difficulty balancing exercise and work demands and incorporating time management strategies to assist participants with integrating exercise into a busy schedule.

The second hypothesis suggested no difference in exercise group sizes between older and middle-aged adults corroborating that item 22 measures the construct it claims. Although previous papers suggested that older adults prefer to exercise alone rather than in a group-based setting, recent findings challenge that literature, and new studies have found older adults prefer group-related interventions among people their own age [17]. One reason why older adults may have suggested solitary exercise programs in previous literature is their perceived view that exercise classes tend to be populated by individuals younger than them [17]. Beauchamp et al. (2007) found older adults prefer exercising in a group setting with individuals their own age [17] and adherence levels tend to be far superior when done in groups compared to alone [25–27]. Future exercise designs should use this item to determine group size preferences for an exercise program and based on the majority, design an exercise program where participants either exercise alone or with other individuals. Since older adults prefer to exercise with people their own age, having an instructor of a similar age to the participants may also help participants feel more comfortable to exercise.

The inverse relationship between SES and physical inactivity has been well demonstrated empirically in the literature [15, 16, 18, 19, 28, 29]. We hypothesized that participants from a lower SES would report cost as a barrier, however, found no association between these two groups. Although the hypothesis was not validated in this study, we doubt the item itself is flawed. Recently, three large systematic reviews emerged questioning this relationship [30–32]. In these reviews, both higher and lower SES groups reported being physically active but the higher SES group was more likely to report leisure-time physical activities such as going to the gym [30] while those in the lower bracket reported housing or occupational physical activities such as cleaning or construction work [31]. Taken together, it is possible that neither the item nor the hypothesis are unreliable since the type of physical activity was not specified. In addition, none of the systematic reviews were able to claim that individuals of higher SES are more active than those in the lower group. More than half of the participants were retired or not working due to disability and reported an income less than $50,000. After removing the retired respondents from the known-group validity test, there were still no differences between groups. Other possible explanations may be that social supports available through the Canadian government for low-income families can reduce the burden of access to exercise facilities and alleviate some of the costs regarding exercise programs. This is still an important item to evaluate and researchers and clinicians should be aware of subsidies that can influence financial costs of an exercise program.

Environmental correlates of physical activity have gained attention over the last decade and include accessibility to a facility, aesthetic attributes, and safety features [15]. The validity of this item is important since the results provide evidence that the item measures what it is supposed to. Environment is hypothesized to influence behavioural intentions based on a meta-analysis that found individuals with a more positive attitude toward their environmental surroundings were more likely to accomplish their intended behaviour [33]. Thus, environmental barriers should not be ignored when designing future exercise programs and promoting adherence. Designing exercise facilities that are safe and aesthetically pleasing may be a simple way to encourage exercise behaviours and the PEQ can be used to identify this.

The PEQ demonstrates moderate test-retest reliability with some domains having better reliability than others. Although some items had a low kappa score this does not necessarily indicate a low confidence rating in the item if it has a high absolute agreement score. An item’s reliability may be questioned when both the absolute agreement and the kappa score are low. Interestingly, even though the test-retest setting was different, where the first survey was completed in the clinic and the second at home, most items demonstrated a moderate to high reliability.

Questions 2 (healthcare’s attitude toward exercise) and 3 (friends/families attitude toward exercise) had the lowest scores in the first domain, which might indicate a hidden problem. It has been reported that 79% of Canadians see a physician more frequently than any other healthcare provider, however, physicians and nurses have the least knowledge and confidence regarding exercise and exercise prescriptions compared to other healthcare provider [34]. Although physicians may want to encourage an active lifestyle, their lack of knowledge and confidence to prescribe exercise may have been reflected in the respondents’ answers. About 28% of participants selected a different answer the second time and there was no pattern to the selection process; a few participants selected “not sure” the first time and “yes” the second, while others selected “yes” the first time and “no” the second. A similar situation may be happening with the respondents’ family and friends. Participants’ family and friends may also believe exercise is important, but may fail to convincingly persuade active participation in exercise.

Questions 4 and 5 regarding the location of an exercise facility and transportation demonstrated “no agreement” and “slight agreement”, respectively. In question 4, the absolute agreement calculation showed 98% of participants selected the same answer in both rounds and the reason for the discrepancy between the unadjusted level of agreement and kappa may be known as the Kappa Paradox. In this paradox, analysis may show a high value for the absolute agreement and a drastically low kappa score [35]. Although a maximum attainable kappa (k_m_) is suggested to fix this imbalance, it may not solve the paradox [35]. Thus, even though question 4 has a low kappa, this does not represent the true precision of the item. Item 5 also demonstrated low reliability. The absolute agreement calculation showed 77% of respondents selected the same answer in both rounds. This item may be indicating that transportation needs fluctuate on a daily bases. The majority of respondents were over the age of 60 and depend on family or friends to assist them. Transportation has been listed as one of the major barriers to exercise in older adults and in the osteoporosis population [36, 37]. Although the reliability of this question is low, it is important to examine the dynamics of this barrier.

Weighted kappa was used to determine the reliability of each item in section 3, which ranged from fair to almost perfect agreement. The lowest subscale scores were in questions 11 (able to walk longer) and 12 (more flexible). Participants may have had more time to think about their goals and reflect on each item since the second questionnaire was completed at home. Older adults leave, rejoin, and switch exercise classes as their commitments and interest change with time and one longitudinal study following 541 participants found 21% dropped out of an exercise program and joined a different program over 3 years [38]. For this reason, exercise goals should be reassessed frequently and individuals should be given the opportunity to try out different programs.

Section four had a reliability score for each item that ranged from moderate to substantial agreement. Question 23 regarding learning proper techniques had the lowest reliability score, which was expected since it had nine options. For this item participants selected one or two more items the second time. Overall, respondents’ answers were not very different from the first round, differing by just one or two choices.

Section five regarding feedback and tracking had the highest reliability, and each item ranged from substantial agreement to almost perfect agreement. Interestingly, the majority of participants that selected “yes” to receiving feedback also selected “yes” to providing feedback and tracking, while the same pattern was seen for those who selected “no”.

The last section, regarding barriers to exercise had a reliability item score that ranged from substantial agreement to almost perfect agreement. There was a general trend where, the second time, participants checked one or two additional barriers. This also could have happened because respondents had more time to think about their barriers while completing the PEQ the second time. .

Although ceiling and flooring effects can be an important consideration for outcome measure questionnaires they are less of a concern for the PEQ since the purpose is to identify the facilitators, barriers, and preferences to exercise. While we were concerned with whether the questionnaire failed to identify these traits, ceiling and floor analyses were not the best way to assess the performance of this type of questionnaire. For example, one barrier is not necessarily a floor effect if it prevents the person from exercising. Similarly, one significant facilitator may offset many smaller barriers, so, for this reason, ceiling and flooring effects would be difficult to interpret. While it may be mathematically possible to calculate ceiling and flooring effects, its interpretation may not be clinically significant.

Despite the substantial work done to validate the PEQ, its usefulness as a tool to devise facilitators, barriers, and preferences to exercise still needs more evaluation. A limitation of this study is that we only evaluated construct validity of 4 items, and so, these results cannot be assumed to generalize other items, although not all items are appropriate for known-group analysis. The next step should test the validity of the remaining questions in the osteoporosis population. One method to test validity is to use a subclass of construct validity such as convergent or discriminant validity. For example, convergent validity for questions 2 (healthcare attitude toward exercise) and 3 (family/friends attitude toward exercise) can be validated with the normative beliefs domain in the Theory of Planned Behaviour Questionnaire. Similarly, entire sections such as domain 3 (my exercise goals) can be validated with the Goal Content for Exercise Questionnaire and question 32 (“I do not exercise as often as I like because:”) and 35 (“do weather conditions stop you from exercising”) can use convergent validity analyses to correlate items on the Self-Efficacy for Exercise Scale. Concurrent validity should not be used to validate the PEQ since this type of validity compares items to a known standard and there are no recognized tools that measure facilitators, barriers, or preferences to exercise in older adults [10].

After confirming the validity of all items in the PEQ, next steps should test this questionnaire in the osteoporosis population and identify some of the major facilitators and barriers and assess different methods to leverage the motivators and limit the obstacles to exercise. Some barriers, such as being in a wheelchair, would require researchers and clinicians to work with their participants to find unique methods to mitigate these barriers in an exercise program. Studies using the PEQ can customize programs and determine its effectiveness to improve exercise adherence in clinical trials. It is also important to train and educate researchers and clinicians how to use the PEQ and help them understand the different factors that affect adherence. In order to see the full benefits of the PEQ, it is important that researchers and clinicians work together with the participants to find solutions to these factors that affect adherence.

### Strengths and limitations

Strengths of this paper include a sample that met sample size calculations, all patients had a diagnosis from a single rheumatologist and a single independent evaluator conducted all the data collection. Although this paper conformed to the highest standards of work, it is not without limitations. Our test-retest sample size was estimated at 46, however only 42 surveys were returned. It is unlikely that 4 more responses would have changed our conclusions, but some imprecision in our estimates is possible.

The PEQ was developed and tested using the southern Ontario population who were mainly Caucasian, so its validity, reliability, and generalizability in other ethnic or religious groups are unknown and geographical factors that affect exercise adherence should also be tested. These issues should be addressed in formal cross-cultural validation studies. This study also recruited more women than men, which could potentially impact the generalizability of the findings to males and many participants were retired or not working due to disability and their reported earnings may have not reflected accurately their true SES. Lastly, we did not collect information on those that declined to participate, which may indicate important differences in their facilitators, barriers, and preferences towards physical activity.

## Conclusion

In this paper, some items in the PEQ demonstrated known-group validity but the remainder still require testing in future studies. The questionnaire also established moderate to high test-retest reliability. The PEQ should be evaluated for additional measurement properties, and most importantly, for its usefulness in exercise prescription and adherence. Implications of this measure could be useful in the development of client-centered exercise interventions for people with low bone mass or osteoporosis.

## Data Availability

The datasets generated and/or analyzed during the current study are not publicly available due to ethics approval and patient confidentiality but are available from the corresponding author on reasonable request.

## Abbreviations

BMD: Bone Mineral Density
I-CVI: Item Content Validity Index
PEQ: Personalized Exercise Questionnaire
PID: Personal Identification Digits
REB: Research Ethics Board
S-CVI: Scale Level Content Validity Index
S-CVI/AVE: Scale Level Content Validity Index Average
S-CVI/UA: Scale Level Content Validity Index Universal Agreement
SES: Socioeconomic Status
